# Supplementary material for: Low RECK Expression Is Part of the Cervical Carcinogenesis Mechanisms
Source: Cancers (Basel). 2021 May 6;13(9):2217. doi: 10.3390/cancers13092217 (PMC8124470; doi:10.3390/cancers13092217)
Supplement: Supplementary file 1 [file cancers-13-02217-s001.zip › cancers-1179866-supplementary.pdf]

Herbster *et al.* 2021 – Figure S1

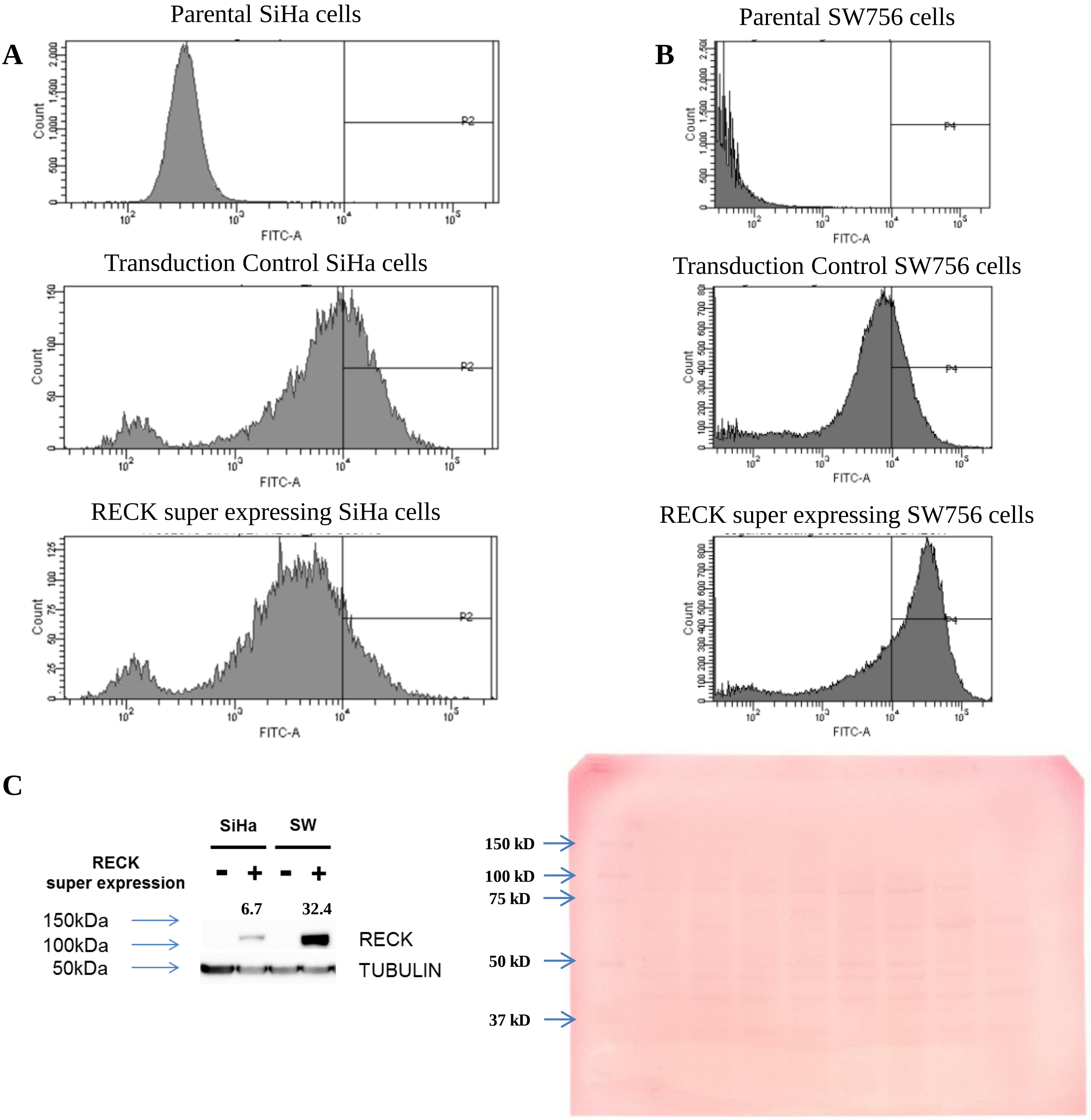

Figure S1. Production of RECK overexpressing cells. A and B. FACS based cell isolation used to enrich for FITC+ SiHa or SW756 populations after transduction. C. RECK protein overexpression was validated by Western blot using 60 micrograms of total protein extracts from lentiviral transduced SiHa and SW756 cell lines. Densitometry data of RECK protein levels in control (-) and RECK (+) overexpressing cells were processed with use of ImageJ software. The PVDF membrane was stained with Ponceau S for load quality control and blue arrows indicate the migration pattern of the protein ladder (far right side). Full length image of RECK and Tubulin immunoblot is below. Only the first four lanes (from left to right) were considered for the densitometry.

Herbster *et al.* 2021 – Figure S1

C

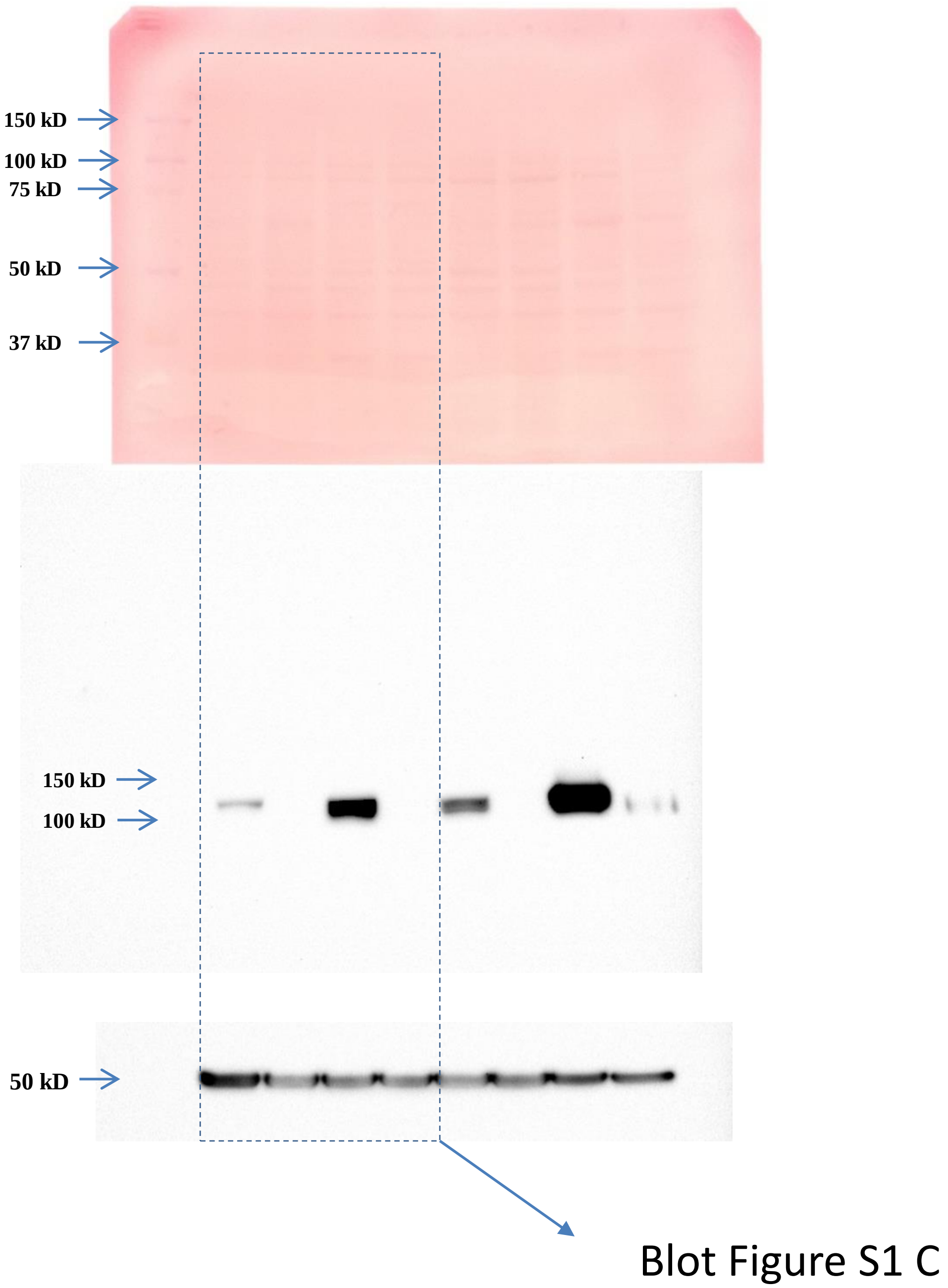

Figure S1. Production of RECK overexpressing cells. A and B. FACS based cell isolation used to enrich for FITC+ SiHa or SW756 populations after transduction. C. RECK protein overexpression was validated by Western blot using 60 micrograms of total protein extracts from lentiviral transduced SiHa and SW756 cell lines. Densitometry data of RECK protein levels in control (-) and RECK (+) overexpressing cells were processed with use of ImageJ software. The PVDF membrane was stained with Ponceau S for load quality control and blue arrows indicate the migration pattern of the protein ladder. Full length image of RECK and Tubulin immunoblot and white light capture of the membrane is below. Only the first four lanes (from left to right) were considered for the densitometry.

Herbster *et al.* 2021 – Table S1

Table S1: List of antibodies (Ab) used for cell labelling prior to flow cytometry analysis.

Ab-target	Ab-clone	Ab-fluorophore	Manufacturer
CD31	ZEH01	APC-Cy7	R&D Systems
CD45.2	104	APC	BD Biosciences
Ly6C	HK1.4	PE	eBiosciences
Ly6G	1A8	PE-Cy7	BD Biosciences
F4/80	BM8	PE-Cy5	eBiosciences
CD49b	DX5	APC-Cy7	BD Biosciences

Herbster *et al.* 2021 – Figure S2

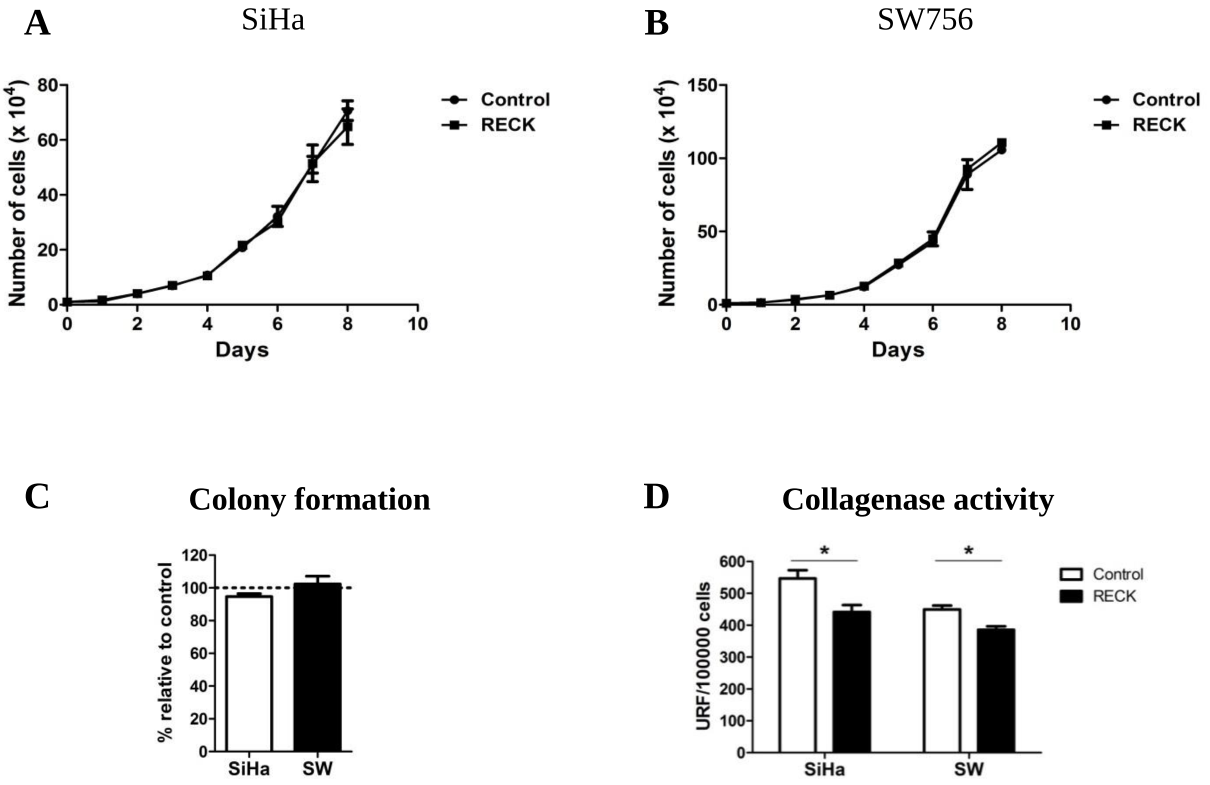

Figure S2. *In vitro* analysis of RECK overexpressing cells. A and B. Monolayer growth potential was determined by growth curves. Cells (2 x 104/well) were seeded in 24 well plates and counted every 24h for eight days. CDT was calculated using log growth phase. C. Cells (1 x 102/well) were seeded in 6 well plates and colony formation was measured after 15 days of culture. Cells were fixed and stained with crystal violet for colony visualization. D. Collagenase activity measured in the supernatant collected from monolayer cultures of SiHa and SW756 control and RECK+. The activity was determined by Collagen IV DQ TM degradation and fluorescent signal measurement with a fluorimeter (510-515 nm filter).

Herbster *et al.* 2021 – Figure S3

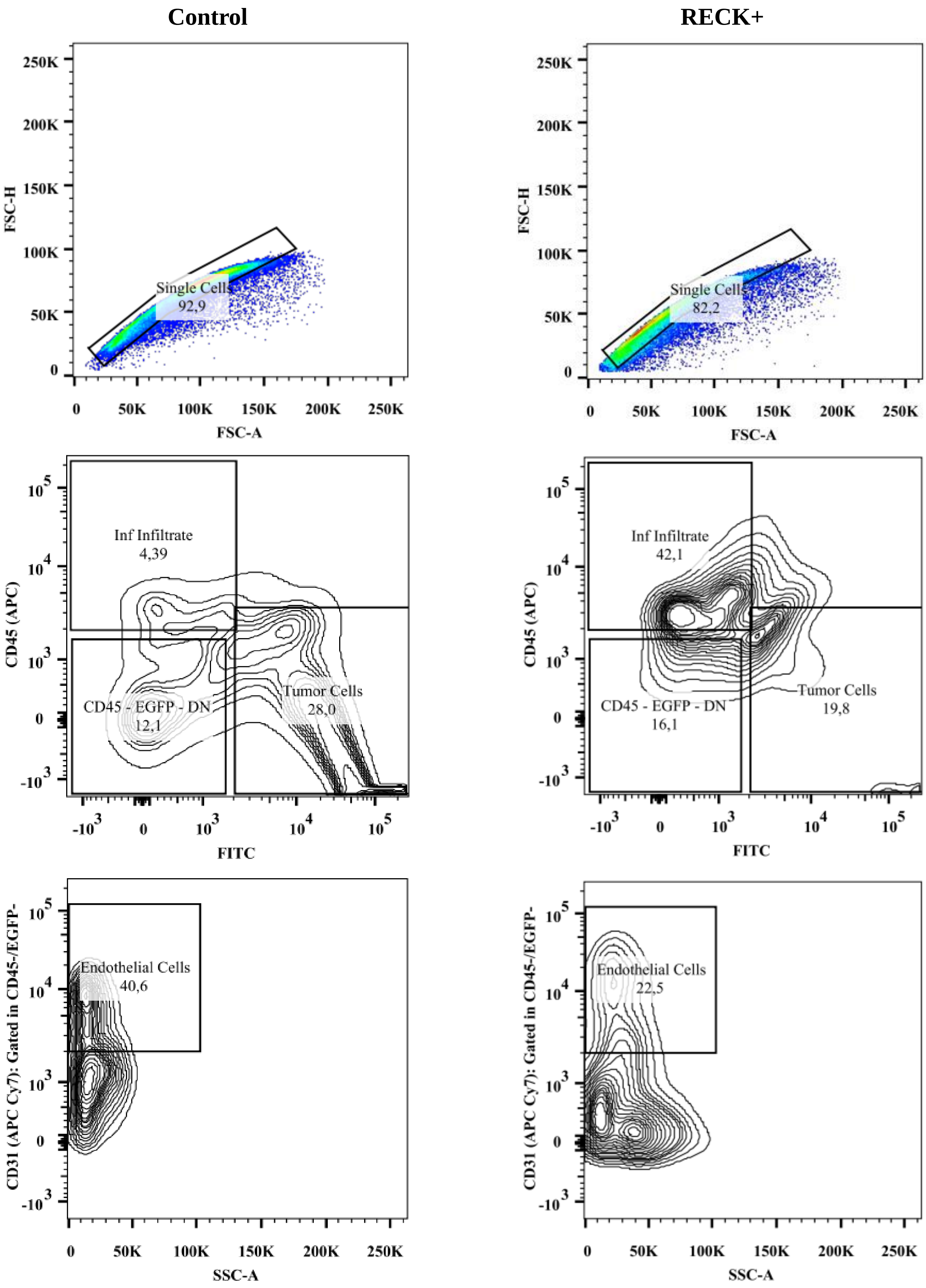

Figure S3. Flow cytometry parameters applied to identify the inflammatory infiltrate and endothelial cells in RECK+ tumors. A. Flow cytometry analysis with intra-tumoral cells isolated from SW756 control and RECK+ tumors. Inflammatory infiltrate and endothelial cells were identified using CD45 and CD31 antibodies, respectively.

Herbster *et al.* 2021 – Figure S4

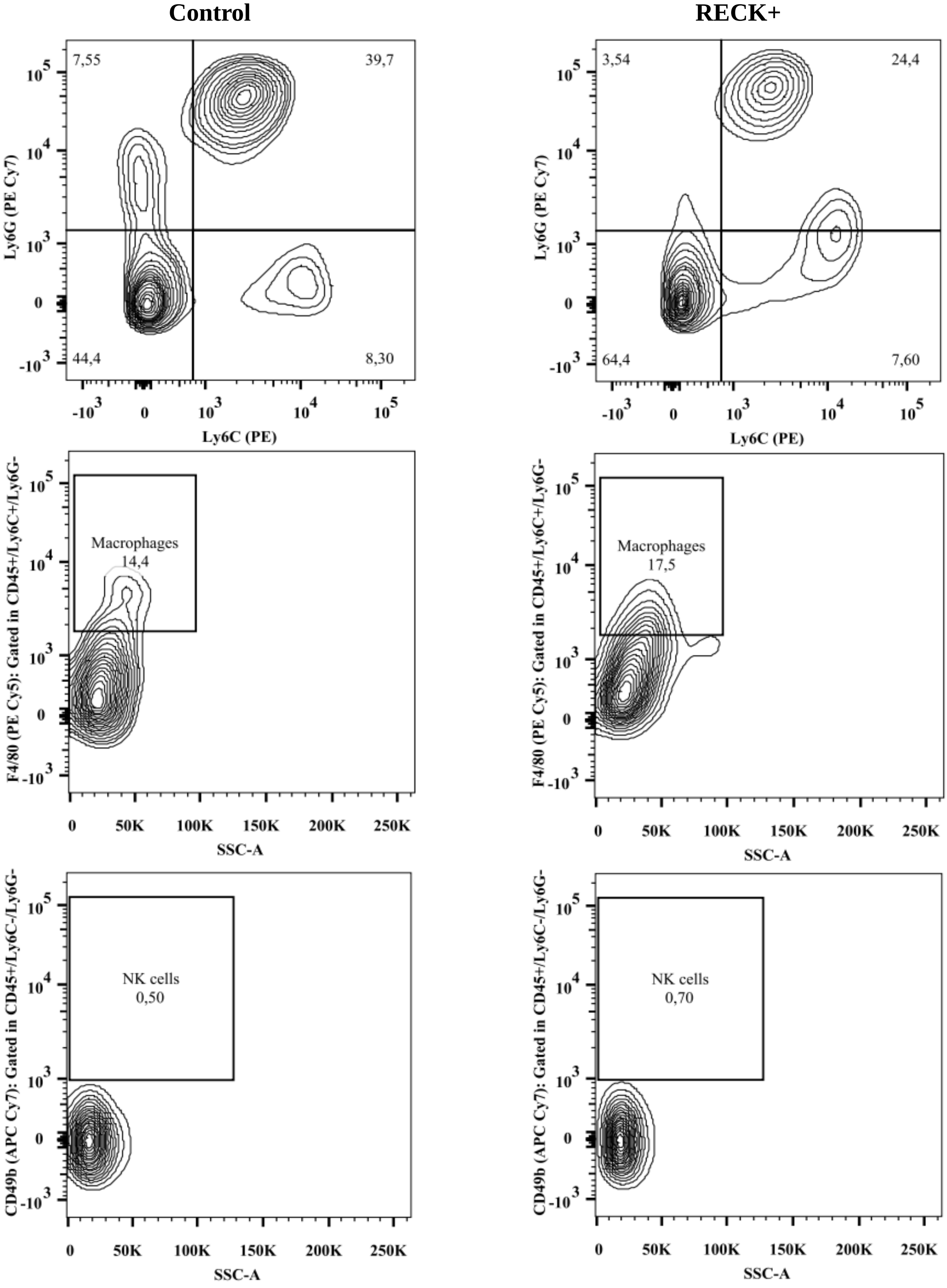

Figure S4. Flow cytometry parameters applied to identify cell populations within the inflammatory infiltrate in RECK+ tumors. A. Flow cytometry analysis with inflammatory cells isolated from SW756 control and RECK+ tumors (Gate: CD45+/EGFP-) isolated from SW756 control and RECK+ tumors. Neutrophils (Ly6G+), Macrophages (Ly6C+/F4/80+), potential MDSC (Ly6C+/Ly6G+), double negative cells (Ly6C-/Ly6G-) and NK cells (Ly6C-/Ly6G-/CD49b+) were identified using specific antibodies.

Herbster *et al.* 2021 – Figure S5

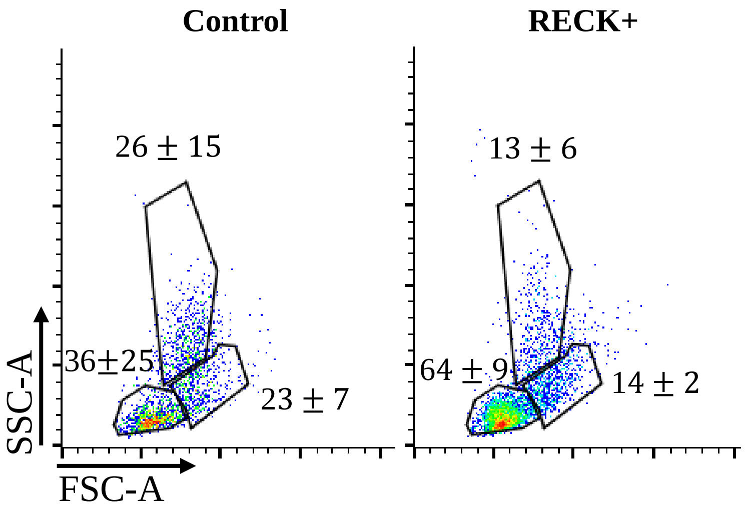

Figure S5. Flow cytometry analysis on FSC vs. SSC parameters of inflammatory cells (Gate:CD45+/EGFP-) in RECK+ tumors. Flow cytometry analysis with inflammatory cells isolated from SW756 control and RECK+ tumors.

Herbster *et al.* 2021 – Figure S6

A **SW756 Control**

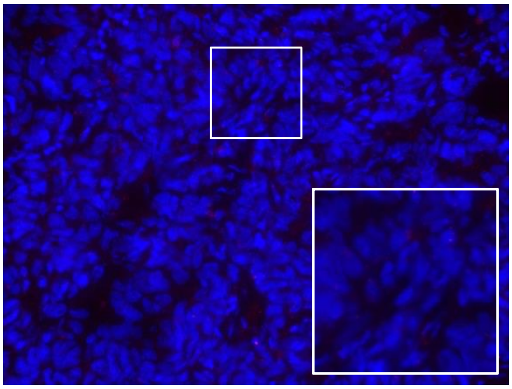

B **SW756 RECK+**

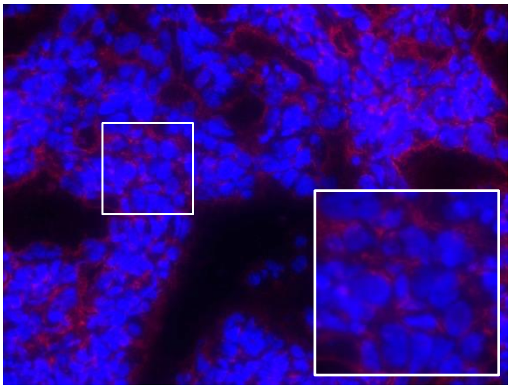

C **RECK immunofluor**

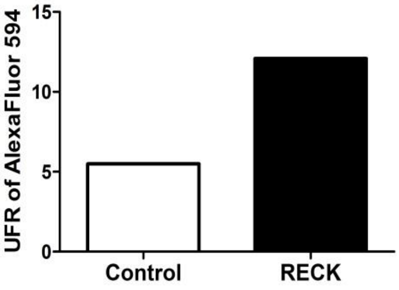

D **In situ zymography**

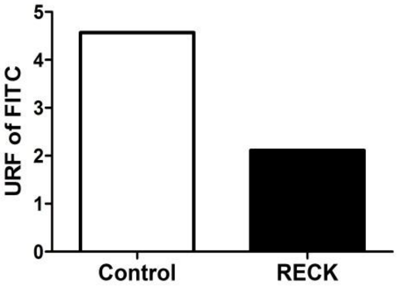

E **Picro Sirius fluor**

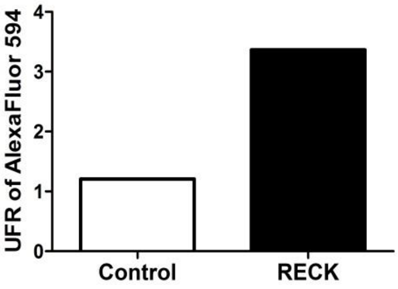

Figure S6. Immunofluorescence assays. A and B. Immunofluorescence assay to detect RECK protein expression (red) in SW756 tumors using specific monoclonal anti-RECK antibody. Nuclei were stained with DAPI (blue). C-E. Analysis of fluorescence emission using ImageJ pixel quantification.

Herbster *et al.* 2021 – Table S2

Table S2: Differentially expressed proteins in SW756 RECK+ tumors versus controls generated by Protein Arrays analysis.

PROTEIN ARRAY R&D	PROTEIN ID	EXPRESSION RECK+ vs. CONTROL	FOLD CHANGE	EXPOSURE TIME (ARRAY POSITION)	CORRELATION WITH RECK mRNA EXPRESSION IN CERVICAL CANCER SAMPLES FROM TCGA
HUMAN ANGIOGENESIS	FGF2	HIGH	1,75	10' (B19,B20)	YES
	AMPHIREGULIN	HIGH	5,35	30' (A17, A18)	-
MOUSE ANGIOGENESIS	ENDOSTATIN/COLLAGEN XVIII	HIGH	3,1	40' (B13, B14)	-
	FGF-BASIC	HIGH	5,2	40' (B19, B20)	YES
	IGFBP-3	LOW	0,2	1'30'' (C17, C18)	-
	MMP-3 PRO AND MATURE FORMS	HIGH	32,5	20' (D15, D16)	-
	MMP-9 PRO AND MATURE FORMS	HIGH	2,1	20' (D19, D20)	-
	PD-ECGF	HIGH	4,7	5' (E3, E4)	-
	PLATELET FACTOR-4	LOW	0,3	20' (E13, E14)	-
	SERPIN-E1	HIGH	2,5	10' (F5, F6)	YES
	THROMBOSPONDIN-2	HIGH	2,8	60' (F9, F10)	YES
PROTEASES	CATHEPSIN-D	LOW	0,1	5' (A17, A18)	-
	CATHEPSIN-B	HIGH	3,4	20' (A13, A14)	-
	CATHEPSIN-E	HIGH	2,4	60' (B3, B4)	-
	CATHEPSIN-L	HIGH	10,7	60' (B5, B6)	-
	CATHEPSIN-S	HIGH	17,1	40' (B7, B8)	-
	CATHEPSIN-V	HIGH	10,0	40' (B9, B10)	-
	CATHEPSIN-X/Z/P	LOW	0,5	10' (B11, B12)	-
	MMP-8	HIGH	2,6	60' (D5, D6)	-
PROTEASES INHIBITORS	CYSTATIN-B	HIGH	1,6	1'30'' (A7, A8)	-
	CYSTATIN-C	HIGH	3,1	5' (A9, A10)	-
	SERPIN-B5	HIGH	3,8	40' (C7, C8)	-
	SERPIN-B6	HIGH	2,4	10' (C9, C10)	-
	SERPIN-B8	HIGH	1,8	1'30'' (C11, C12)	-
	SERPIN-E1/PAI-1	HIGH	4,4	10' (C13, C14)	YES
	SERPIN-F1	LOW	0,3	60' (C15, C16)	-
	TESTICAN-1/SPOCK-1	LOW	0,1	60' (C17, C18)	-
	TFPI	LOW	0,2	60' (D5, D6)	-
	TIMP-2	HIGH	2,0	40' (D11, D12)	YES
PHOSPHO-IMMUNORECEPTORS	NKp46/NCR1	HIGH	1,7	60' (E1, E2)	-
SOLUBLE RECEPTORS	ALCAM/CD166	HIGH	3,4	20' (A13, A14)	-
	AMPHIREGULIN	HIGH	2,6	20' (A15, A16)	-
	CD36/CR-B3	HIGH	1,6	40' (B15, B16)	-
	CD40/TNFRSF5	HIGH	3,0	3' (B11, B12)	-
	CD99	HIGH	1,5	20' (C5, C6)	-
	CRELD2	HIGH	4,7	2' (C3, C4)	-
	CXCL8/IL-8	LOW	0,3	10' (C13, C14)	-
	ECM-1	HIGH	10,0	2' (C7, C8)	-
	EPCAM/TROP-1	LOW	0,2	5' (C13, C14)	-
	GALECTIN-3	HIGH	4,1	10' (C23, C24)	-
	INTEGRIN-B2/CD18	HIGH	1,8	20' (D13, D14)	-
	NOTCH-1	LOW	0,4	40' (E9, E10)	-
	PAR-1	HIGH	2,1	10' (E15, E16)	YES
	SYNDECAN-4	HIGH	2,3	40' (F5, F6)	-
	TIMP-2	HIGH	3,6	20' (F5, F6)	YES

Herbster *et al.* 2021 – Figure S7

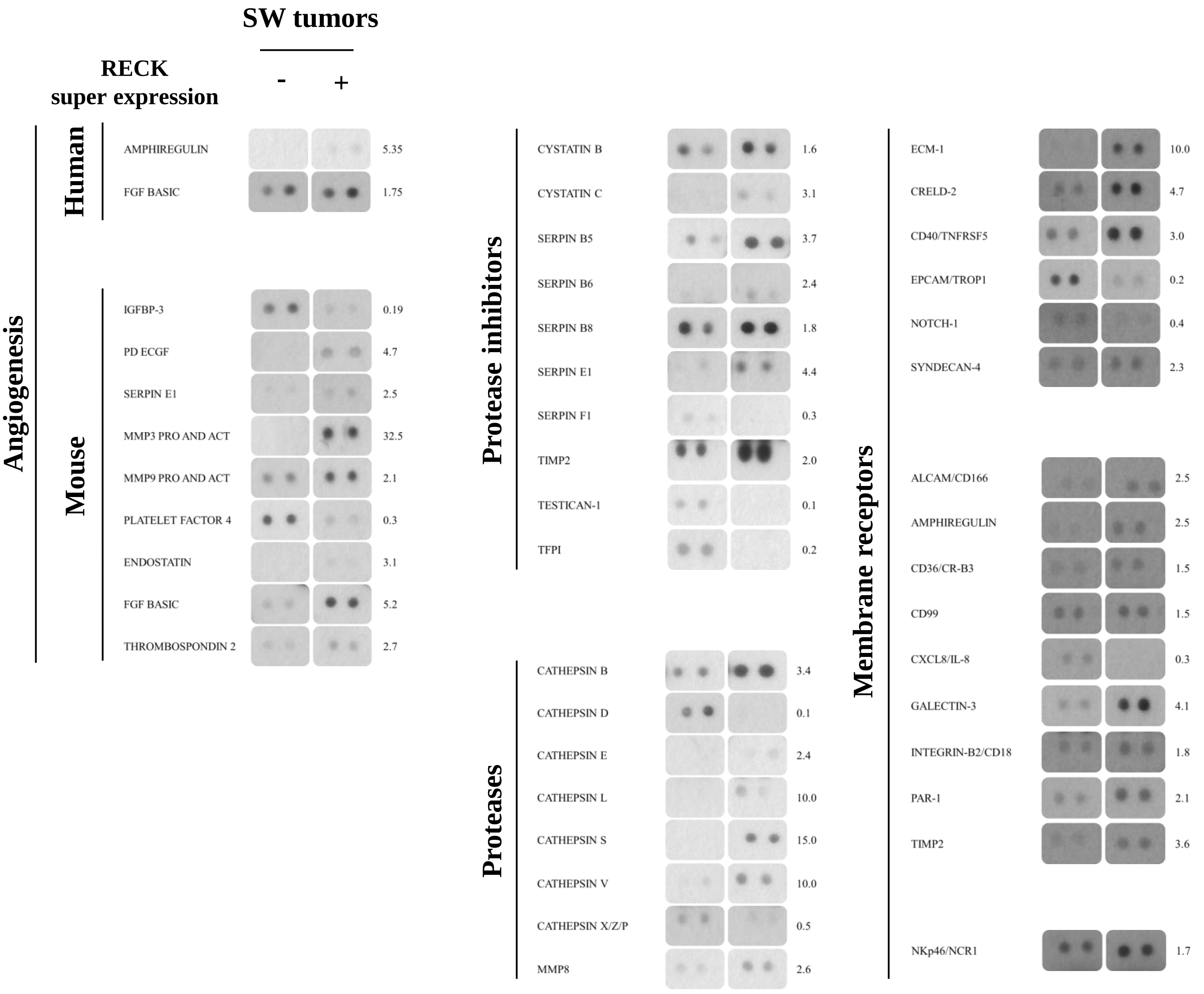

Figure S7. Differentially expressed proteins in SW756 RECK+ tumors versus controls generated by Protein Arrays analysis. Representations of protein arrays data from autoradiography films. Pixel densities on developed X-ray films were acquired with Image J software. The values depicted on the right side of each protein spot represent the fold change expression in SW756 RECK+ vs control tumors.

Herbster *et al.* 2021 – Figure S7

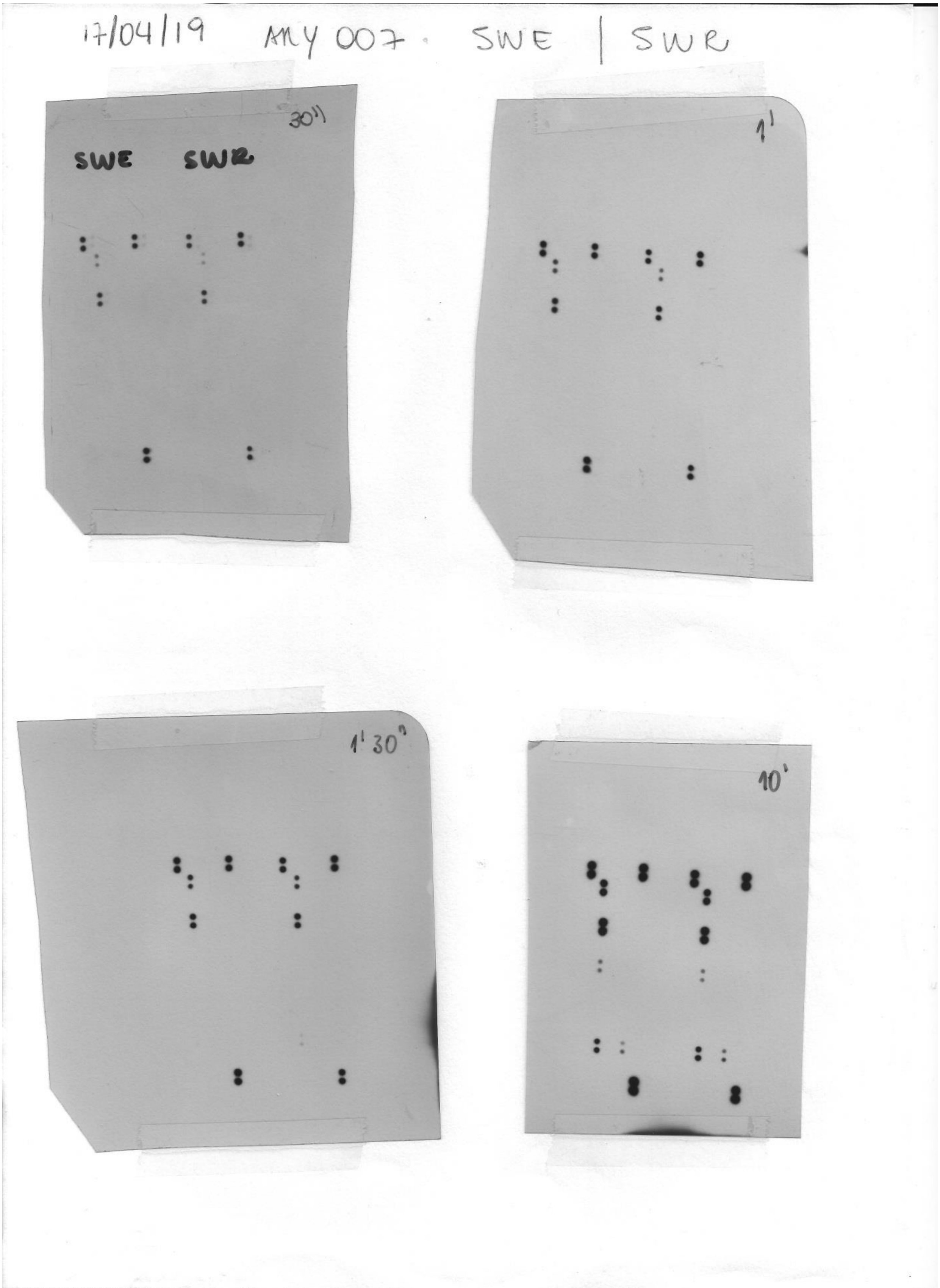

Figure S7. Differentially expressed proteins in SW756 RECK+ tumors versus controls generated by Protein Arrays analysis. Representations of protein arrays data from autoradiography films. Pixel densities on developed X-ray films were acquired with Image J software.

Herbster *et al.* 2021 – Figure S7

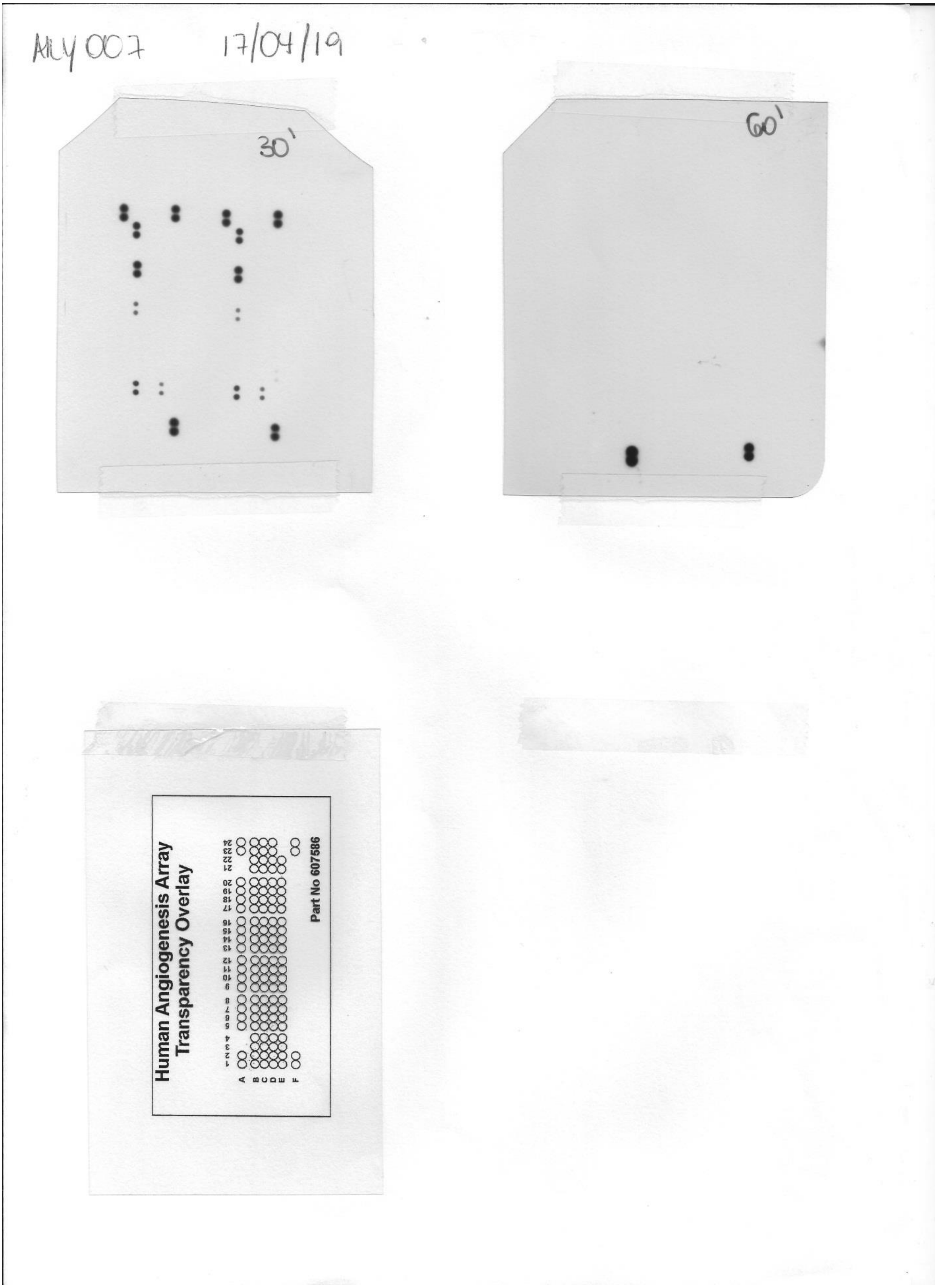

Figure S7. Differentially expressed proteins in SW756 RECK+ tumors versus controls generated by Protein Arrays analysis. Representations of protein arrays data from autoradiography films. Pixel densities on developed X-ray films were acquired with Image J software.

Herbster *et al.* 2021 – Figure S7

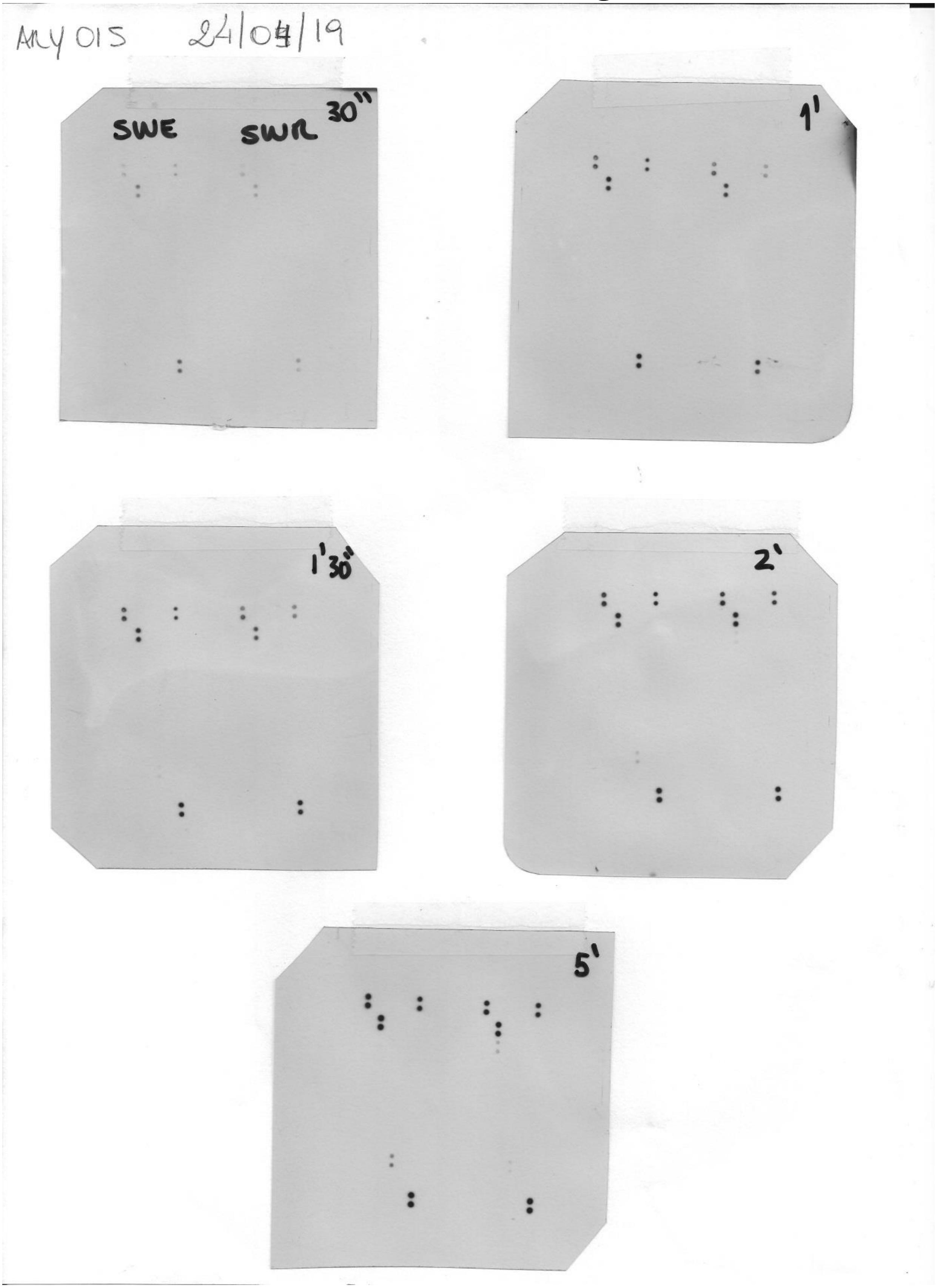

Figure S7. Differentially expressed proteins in SW756 RECK+ tumors versus controls generated by Protein Arrays analysis. Representations of protein arrays data from autoradiography films. Pixel densities on developed X-ray films were acquired with Image J software.

Herbster *et al.* 2021 – Figure S7

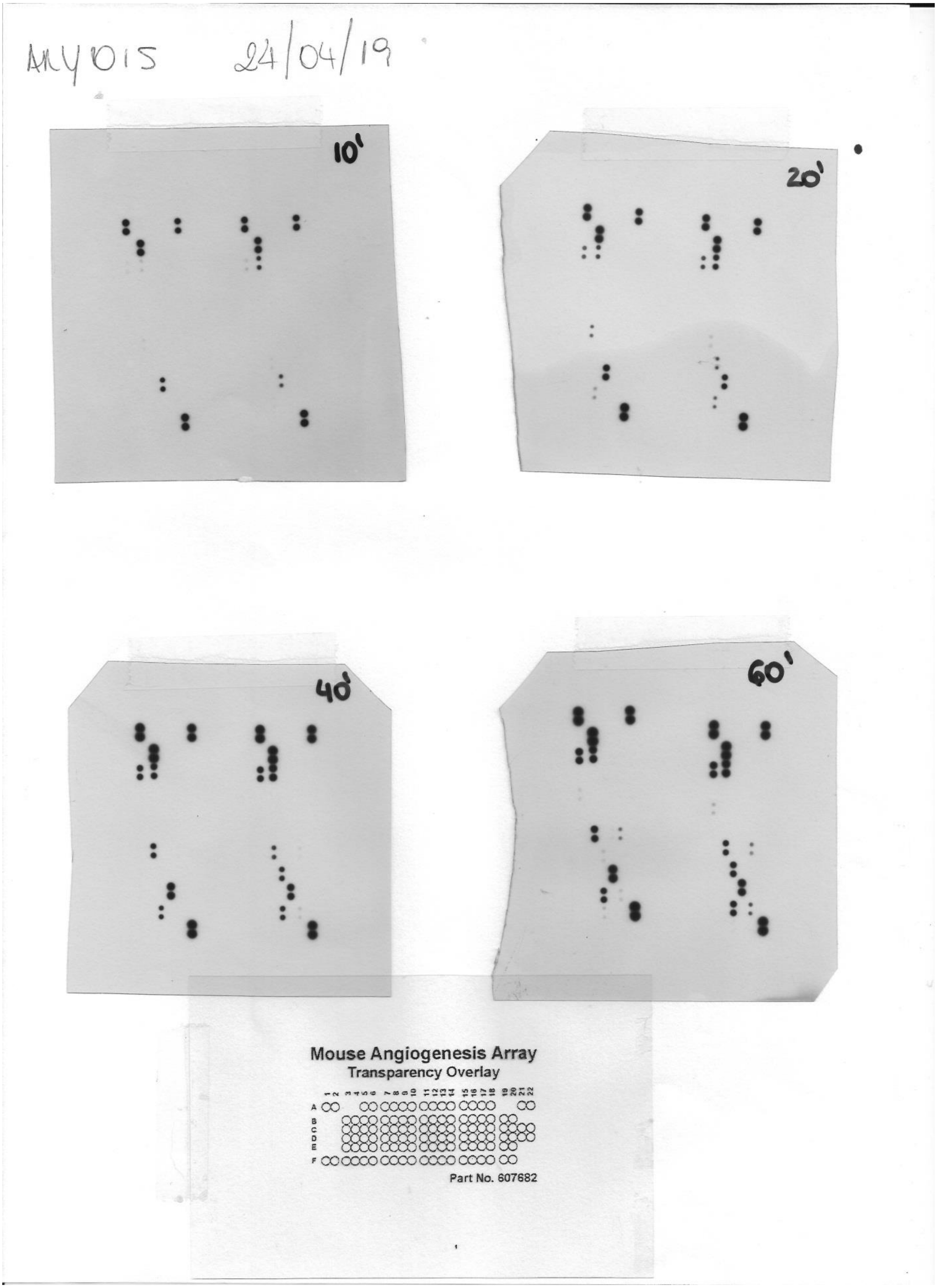

Figure S7. Differentially expressed proteins in SW756 RECK+ tumors versus controls generated by Protein Arrays analysis. Representations of protein arrays data from autoradiography films. Pixel densities on developed X-ray films were acquired with Image J software.

Herbster *et al.* 2021 – Figure S7

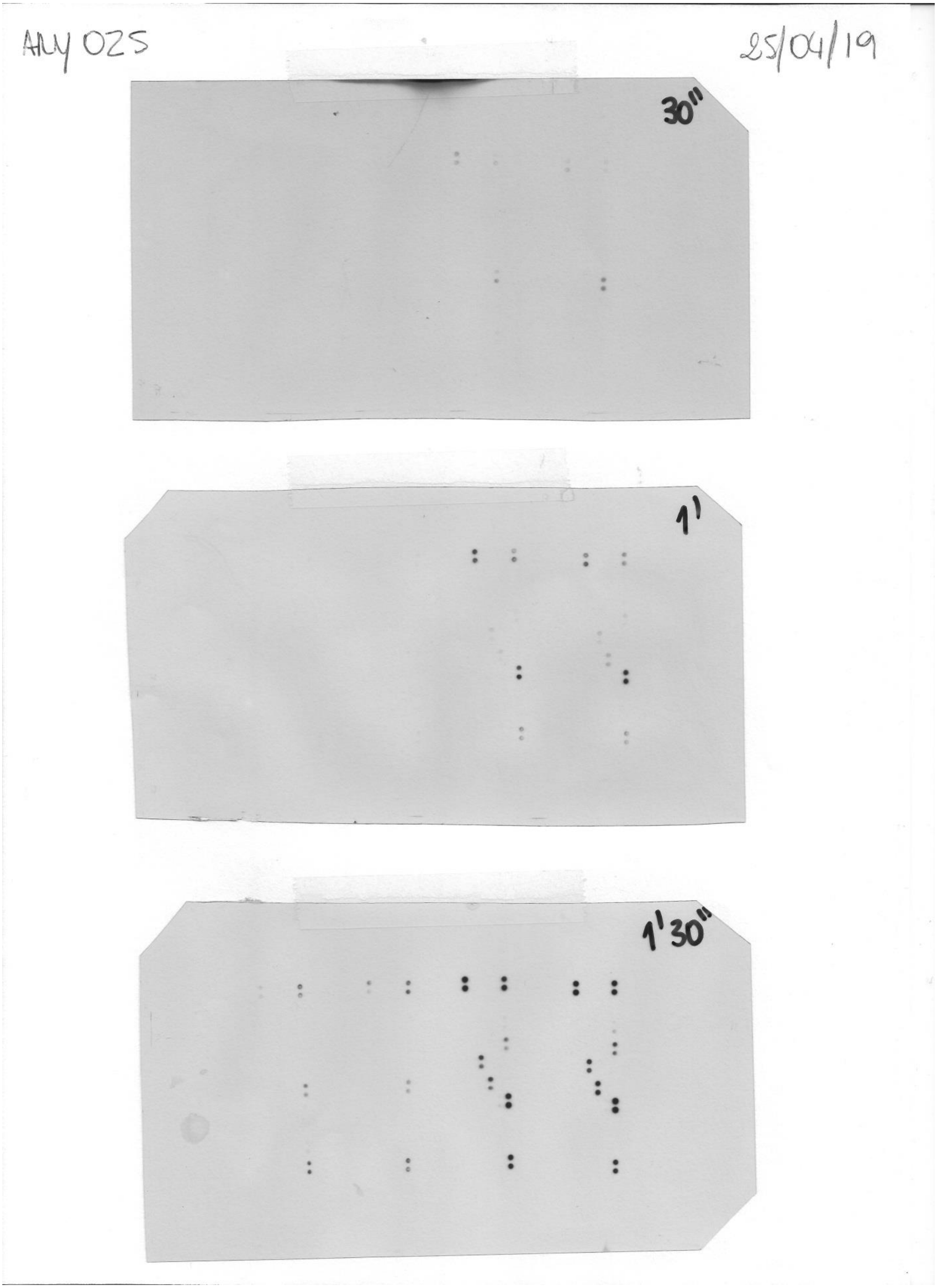

Figure S7. Differentially expressed proteins in SW756 RECK+ tumors versus controls generated by Protein Arrays analysis. Representations of protein arrays data from autoradiography films. Pixel densities on developed X-ray films were acquired with Image J software.

Herbster *et al.* 2021 – Figure S7

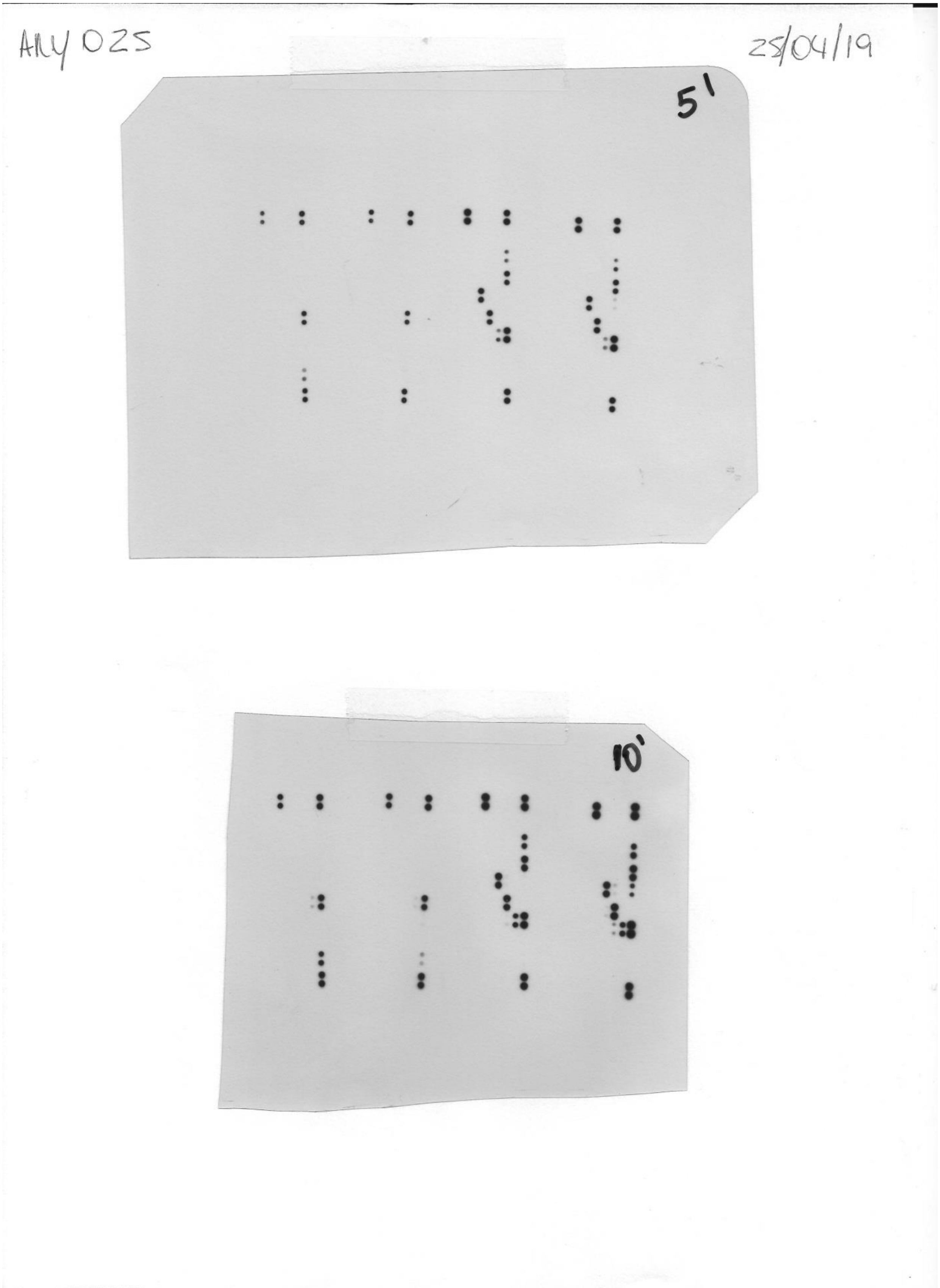

Figure S7. Differentially expressed proteins in SW756 RECK+ tumors versus controls generated by Protein Arrays analysis. Representations of protein arrays data from autoradiography films. Pixel densities on developed X-ray films were acquired with Image J software.

Herbster *et al.* 2021 – Figure S7

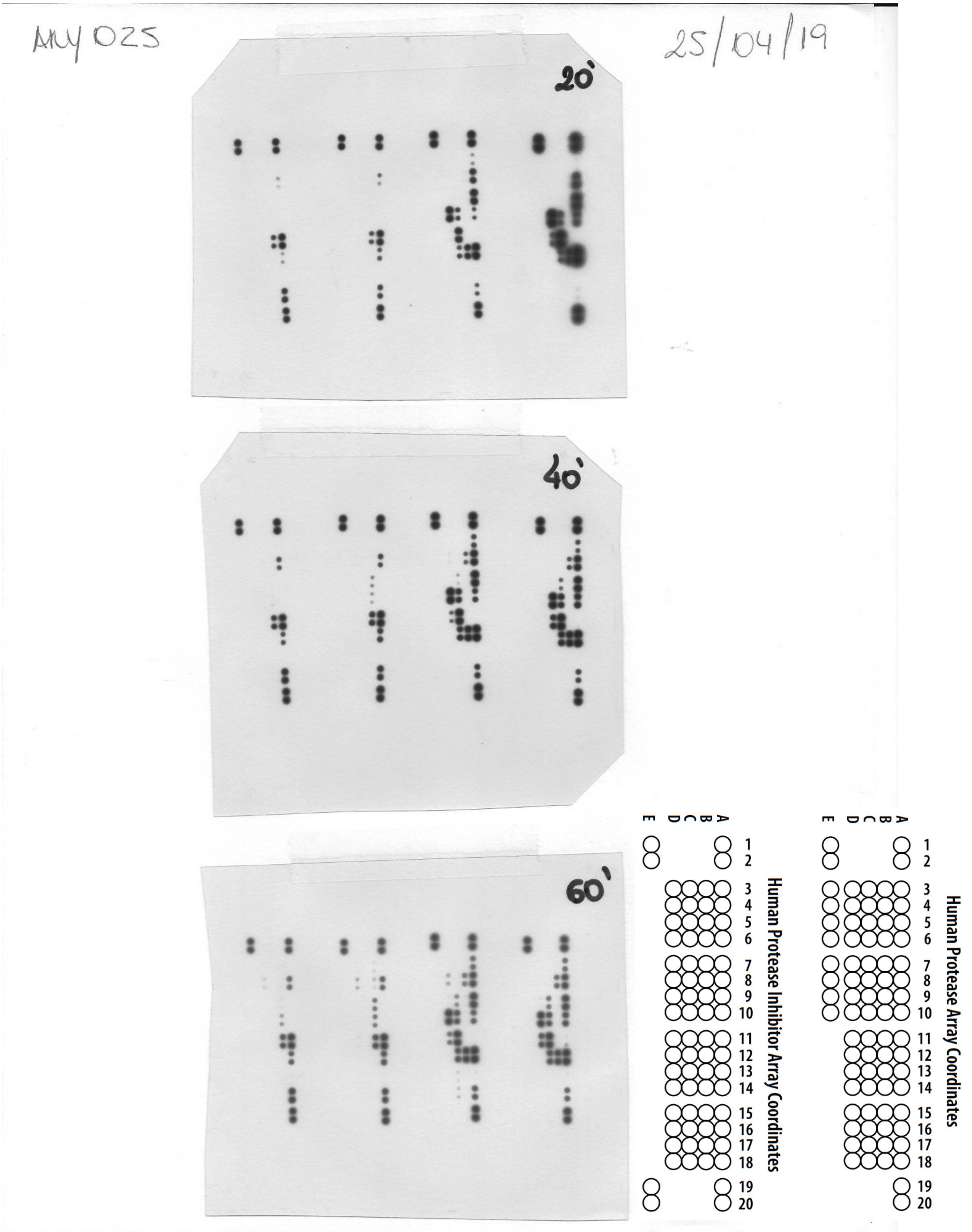

Figure S7. Differentially expressed proteins in SW756 RECK+ tumors versus controls generated by Protein Arrays analysis. Representations of protein arrays data from autoradiography films. Pixel densities on developed X-ray films were acquired with Image J software.

Herbster *et al.* 2021 – Figure S7

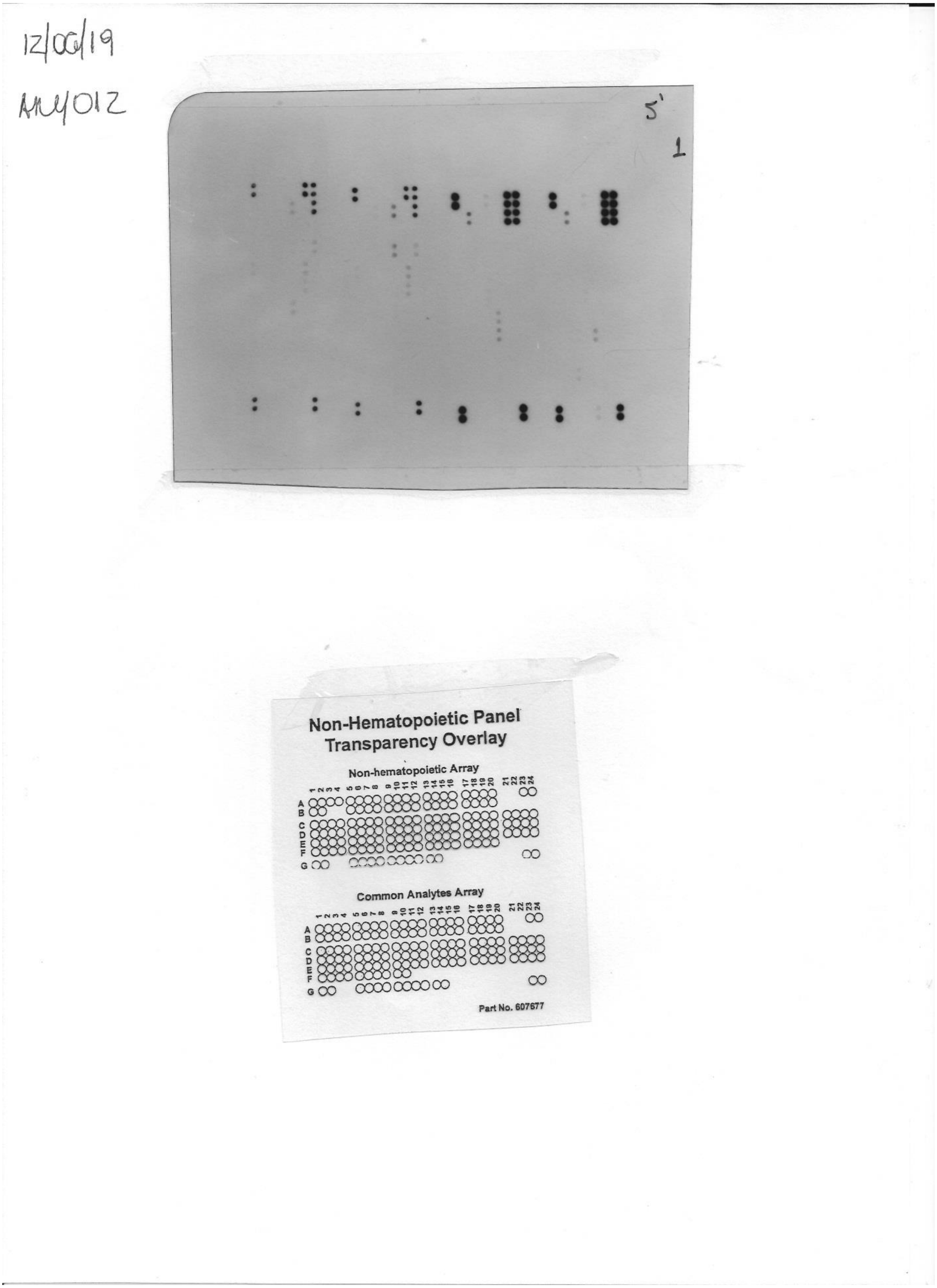

Figure S7. Differentially expressed proteins in SW756 RECK+ tumors versus controls generated by Protein Arrays analysis. Representations of protein arrays data from autoradiography films. Pixel densities on developed X-ray films were acquired with Image J software.

Herbster *et al.* 2021 – Figure S7

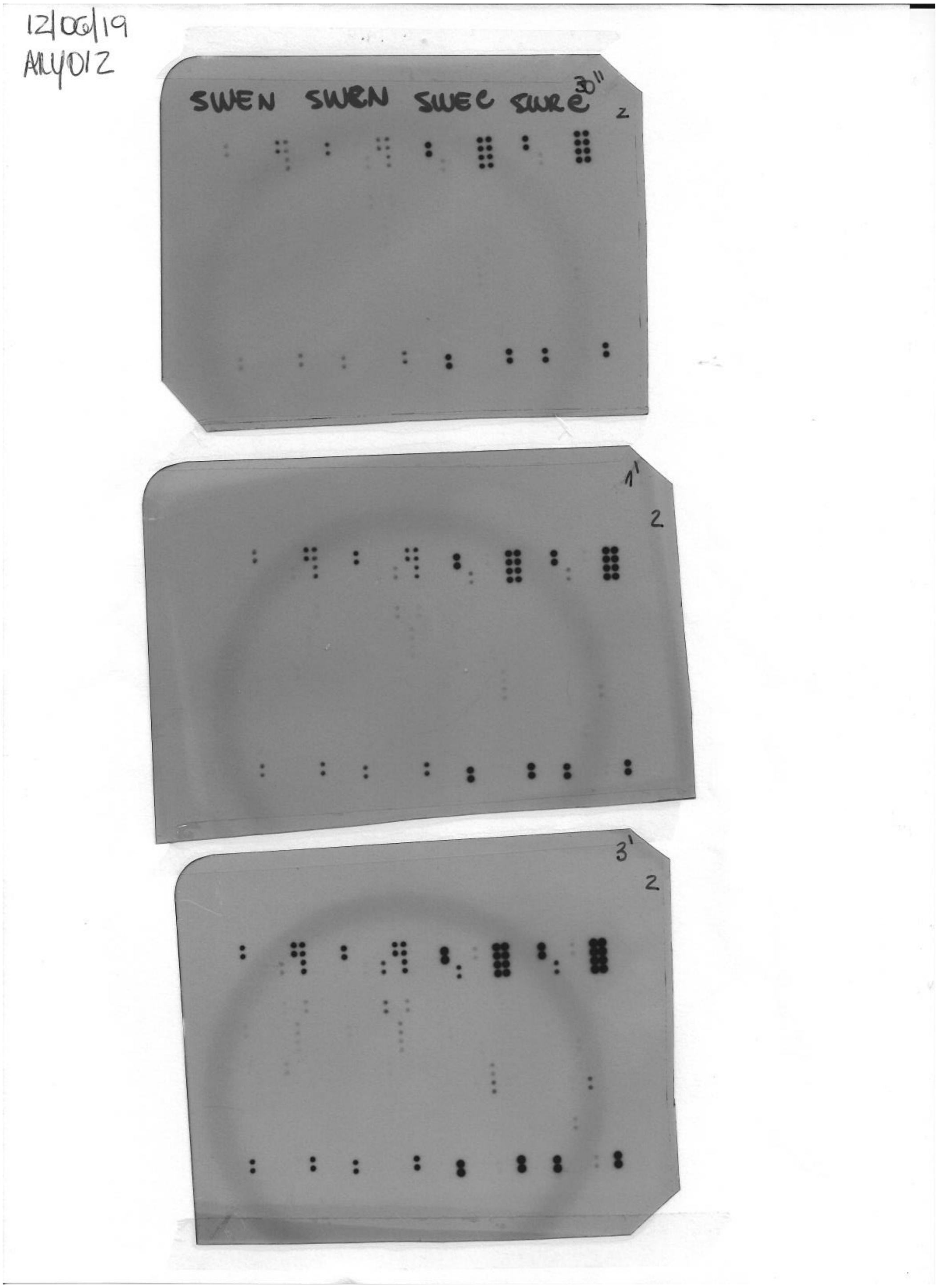

Figure S7. Differentially expressed proteins in SW756 RECK+ tumors versus controls generated by Protein Arrays analysis. Representations of protein arrays data from autoradiography films. Pixel densities on developed X-ray films were acquired with Image J software.

Herbster *et al.* 2021 – Figure S7

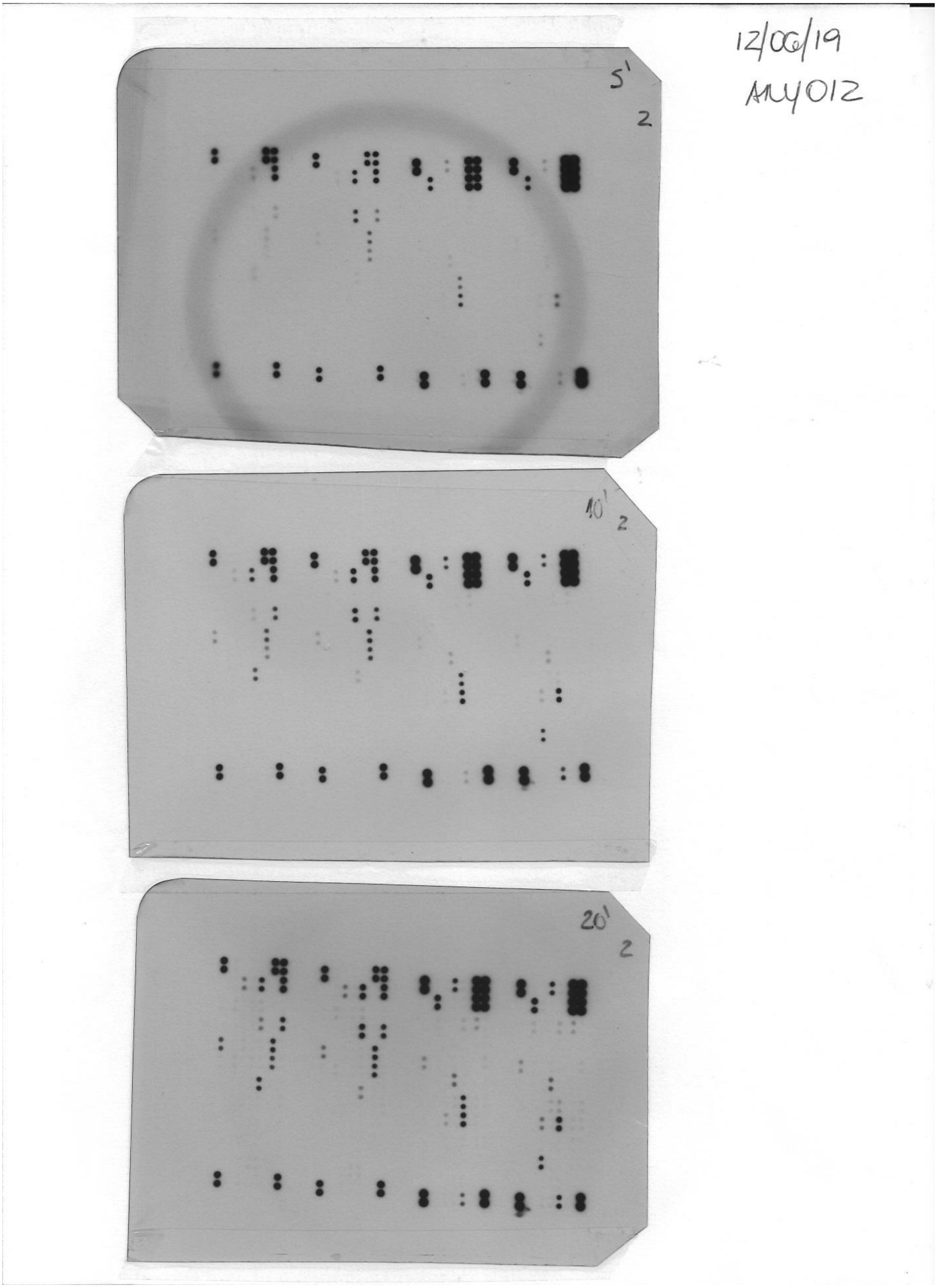

Figure S7. Differentially expressed proteins in SW756 RECK+ tumors versus controls generated by Protein Arrays analysis. Representations of protein arrays data from autoradiography films. Pixel densities on developed X-ray films were acquired with Image J software.

Herbster *et al.* 2021 – Figure S7

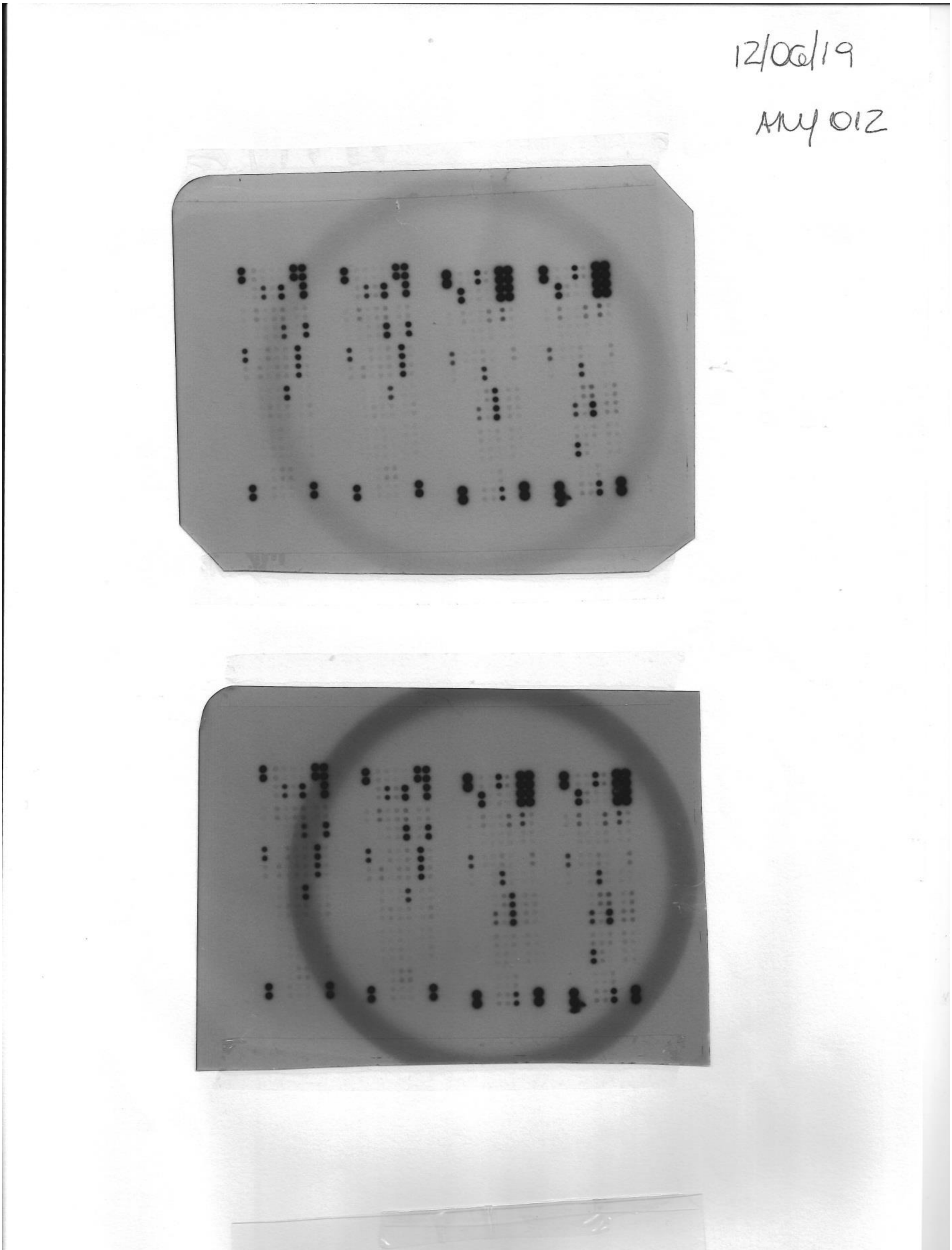

Figure S7. Differentially expressed proteins in SW756 RECK+ tumors versus controls generated by Protein Arrays analysis. Representations of protein arrays data from autoradiography films. Pixel densities on developed X-ray films were acquired with Image J software.

Herbster *et al.* 2021 – Figure S7

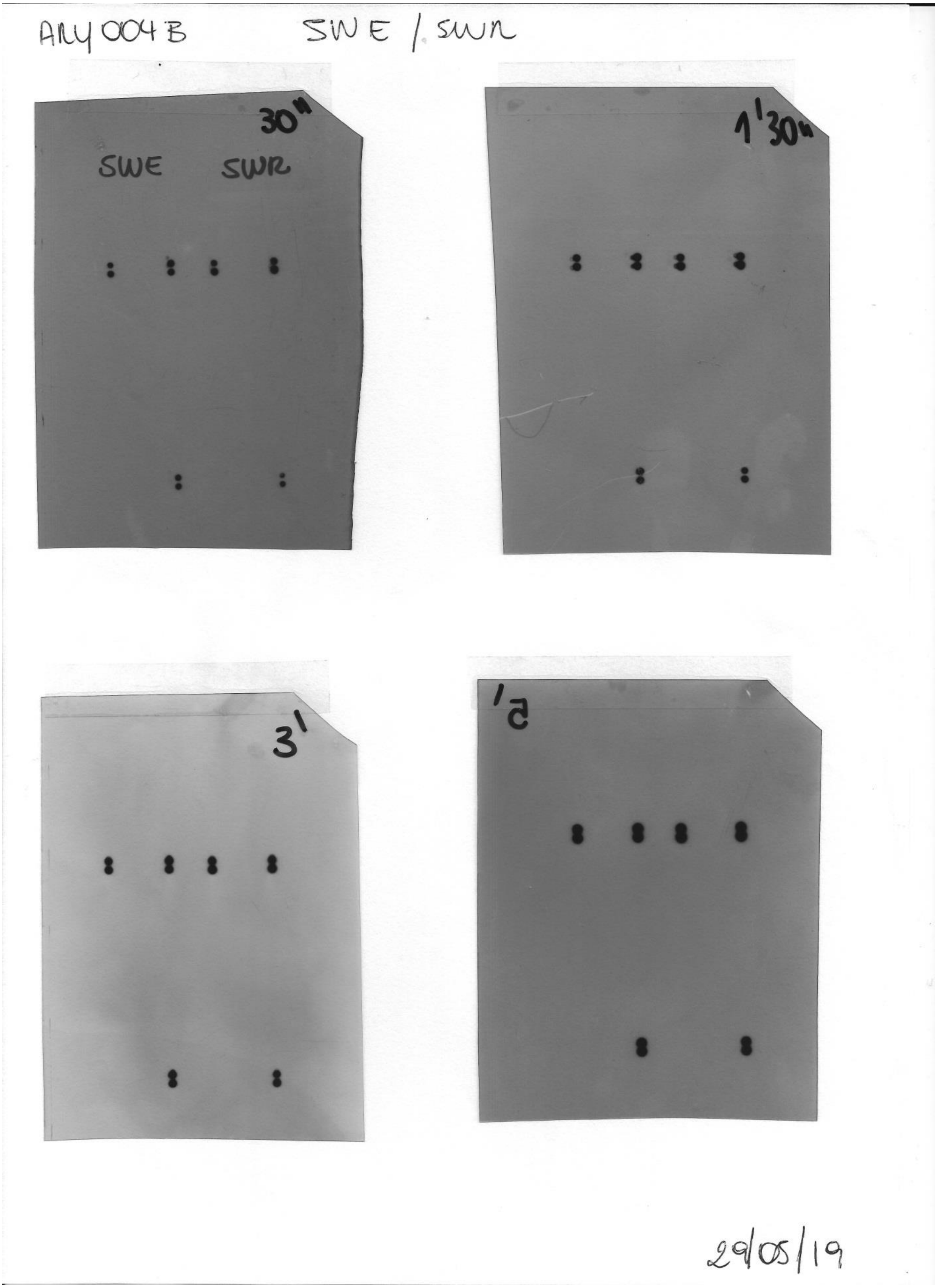

Figure S7. Differentially expressed proteins in SW756 RECK+ tumors versus controls generated by Protein Arrays analysis. Representations of protein arrays data from autoradiography films. Pixel densities on developed X-ray films were acquired with Image J software.

Herbster *et al.* 2021 – Figure S7

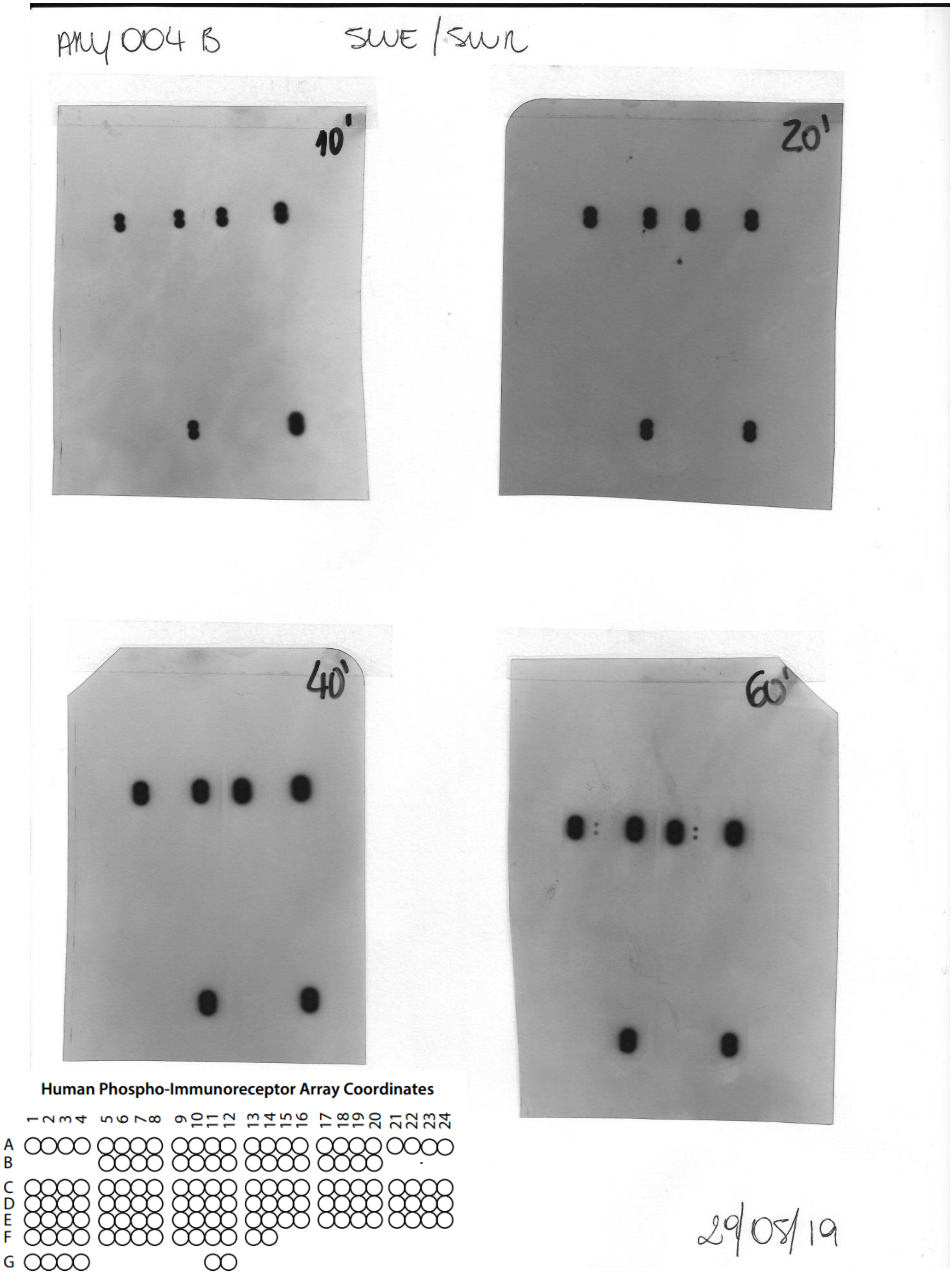

Figure S7. Differentially expressed proteins in SW756 RECK+ tumors versus controls generated by Protein Arrays analysis. Representations of protein arrays data from autoradiography films. Pixel densities on developed X-ray films were acquired with Image J software.

Herbster *et al.* 2021 – Figure S8

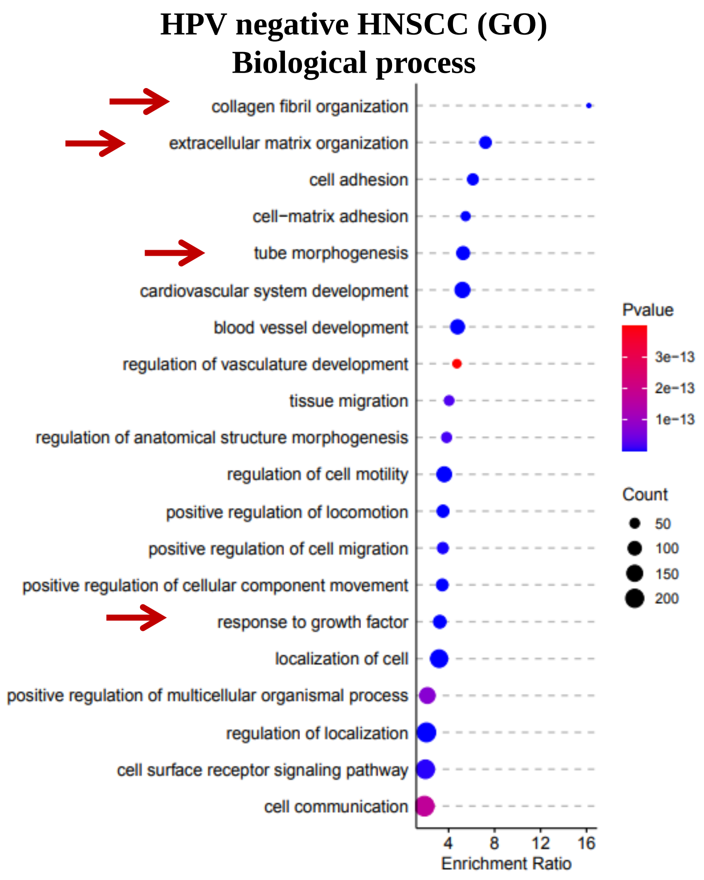

Figure S8. GO pathway enrichment analysis focused on biological processes most associated with RECK expression in HPV negative HNSCC clinical samples.
